# Comparative lipidomics of 5-Fluorouracil–sensitive and –resistant colorectal cancer cells reveals altered sphingomyelin and ceramide controlled by acid sphingomyelinase (SMPD1)

**DOI:** 10.1038/s41598-020-62823-0

**Published:** 2020-04-09

**Authors:** Jae Hun Jung, Kohei Taniguchi, Hyeong Min Lee, Min Young Lee, Raju Bandu, Kazumasa Komura, Kil Yeon Lee, Yukihiro Akao, Kwang Pyo Kim

**Affiliations:** 1 Department of Applied Chemistry, Institute of Natural Science, Global Center for Pharmaceutical Ingredient Materials, Kyung Hee University, Yongin, Republic of Korea; 2 Department of Biomedical Science and Technology, Kyung Hee Medical Science Research Institute, Kyung Hee University, Seoul, Republic of Korea; 3 Translational Research Program, Osaka Medical College, 2-7 Daigaku-machi, Takatsuki, Osaka 569-8686 Japan; 4 Institute for Systems Biology, Seattle, WA USA; 5 Department of Surgery, College of Medicine, Kyung Hee University, Seoul, Republic of Korea; 6 United Graduate School of Drug Discovery and Medical Information Sciences, Gifu University, 1-1 Yanagido, Gifu, 501-1193 Japan

**Keywords:** Lipidomics, Lipids, Proteomics, Biochemistry, Diseases, Cancer

## Abstract

5-Fluorouracil (5-FU) is a chemotherapeutic drug widely used to treat colorectal cancer. 5-FU is known to gradually lose its efficacy in treating colorectal cancer following the acquisition of resistance. We investigated the mechanism of 5-FU resistance using comprehensive lipidomic approaches. We performed lipidomic analysis on 5-FU–resistant (DLD-1/5-FU) and -sensitive (DLD-1) colorectal cancer cells using MALDI-MS and LC-MRM-MS. In particular, sphingomyelin (SM) species were significantly up-regulated in 5-FU–resistant cells in MALDI-TOF analysis. Further, we quantified sphingolipids including SM and Ceramide (Cer) using Multiple Reaction Monitoring (MRM), as they play a vital role in drug resistance. We found that 5-FU resistance in DLD-1/5-FU colorectal cancer cells was mainly associated with SM increase and Cer decrease, which are controlled by acid sphingomyelinase (SMPD1). In addition, reduction of SMPD1 expression was confirmed by LC-MRM-MS analysis and the effect of SMPD1 in drug resistance was assessed by treating DLD-1 cells with siRNA-SMPD1. Furthermore, clinical colorectal cancer data set analysis showed that down-regulation of SMPD1 was associated with resistance to chemotherapy regimens that include 5-FU. Thus, from our study, we propose that SM/Cer and SMPD1 are new potential target molecules for therapeutic strategies to overcome 5-FU resistance.

## Introduction

Colorectal cancer (CRC) is one of the leading causes of cancer-related mortality in both men and women^1^. Although there are other drugs for treatment of CRC, 5-Fluorouracil (5-FU) is widely used and is positioned as a first-line chemotherapy. 5-FU was developed as an inhibitor of thymidylate synthase (TS), which results in suppression of thymine synthase, resulting in cell death^2^. The mechanism involves misincorporation of a pyrimidine analogue into RNA and DNA in place of uracil or thymine, respectively^3^. Despite the effectiveness of 5-FU, drug resistance remains a significant limitation. To overcome this drug resistance, many researchers have tried to identify potential genes and proteins involved in mediating 5-FU resistance, using emerging technologies such as microarray profiling^4^ and whole genome sequencing^5^. For instance, the alteration of drug influx and efflux by the ABCC5 membrane protein and mutation of the drug target^6^ may lead to 5-FU resistance. Furthermore, accumulation of TS protein and elevated activity of deoxyuridine triphosphatase are expected to cause 5-FU resistance in CRC. Although various target genes are involved, the detailed 5-FU-resistance mechanism has not been fully elucidated. Therefore, new strategies for therapy and resistance reversal are urgently needed.

Various lipidomic approaches have revealed that lipids play key roles in various phenomena in living cells including oncogenesis^7,8^, apoptosis^9^, and drug resistance^10–12^. Alterations in levels of glycerophospholipids (GPs) such as phosphatidylcholine (PC) and phosphatidylethanolamine (PE) have been often considered as biochemical indicators of tumor progression or drug response^13,14^. In particular, sphingolipids (SLs) such as sphingomyelin (SM), ceramide (Cer), and sphingosine 1-phosphate (S1P) are known as the central molecules, controlling various aspects of cell growth and proliferation in cancer, and have been implicated in the mechanisms of action of cancer chemotherapeutics^15,16^. A previous study (Chiranjeevi Peetla *et al*.) reported that doxorubicin-resistant (MCF-7/ADR) breast cancer cells showed significant increase in plasma membrane SM, which interacts with cholesterol. This interaction forms a more condensed, solid plasma membrane compared to those of doxorubicin-sensitive cells. The rigidity of the membranes of the resistant cells inhibits drug uptake when using a liposomal formulation of doxorubicin^17^. When Cer is stacked in a lipid raft through breakdown of SM into Cer by acid sphingomyelinase (SMPD1), the death receptor FAS aggregates in the lipid raft, which leads to programmed cell death (apoptosis)^18^. However, defects in Cer and its generation, as well as its metabolism in cancer cells, contribute to tumor cell survival and resistance to chemotherapy. Thus, SMPD1 regulation might be very important in controlling the mechanism of resistance to 5-FU.

Although differences of lipid species between 5-FU-sensitive and -resistant cells are important for the 5-FU resistance mechanism in CRC, there have been few studies using global lipidomic analysis of 5-FU–resistant CRC. In the current study, GPs and SLs associated with 5-FU resistance in CRC were successfully identified and quantified using MALDI-MS and LC-MRM-MS approaches. Of note, SL species and proteins involved in the pathway of SL metabolism were quantified by accurate multiple reaction monitoring (MRM) to understand the relationship between 5-FU resistance and the SL pathway. A decrease of SMPD1 protein with a significant increase of SM and a decrease of Cer has been proven to be a very important mechanism in 5-FU resistance. Furthermore, using Oncomine data set analysis, we investigated the expression level of mRNA of SMPD1 in CRC clinical samples in response to 5-FU treatment. Together, our findings propose a novel mechanism of 5-FU resistance and an effective cancer therapy by combining drugs that target angiogenesis and lipid metabolism.

## Results

### Profiling and semi quantification of lipids by MALDI-MS analysis

**Figure 1:**
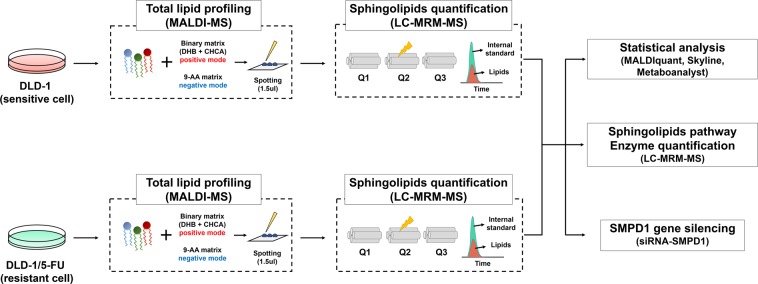
Schematic diagram of global lipidomic analysis. Total lipids were extracted from 5-FU–sensitive (DLD-1) and –resistant (DLD-1/5-FU) colorectal cancer cells and subjected to MALDI-MS analysis in positive and negative modes in triplicate. MALDI-MS spectra were processed by MALDI-Quant in R package. The transition of sphingolipid and related enzymes (Q1 and Q3) was optimized and quantified using MRM-based LC-QqQ-MS analysis in triplicate. Statistical analysis of quantified lipids was carried out using Metaboanalyst.

To investigate global alterations in cell lipid composition, we used the parental DLD-1 cell line, which is a human colorectal adenocarcinoma cell line, and its 5-FU-resistant version DLD-1/5-FU. Extracted lipids were analyzed by MALDI-MS in both positive and negative ion modes (see the Methods section for details). Figure 1 shows the detailed workflow of the present study.

**Figure 2:**
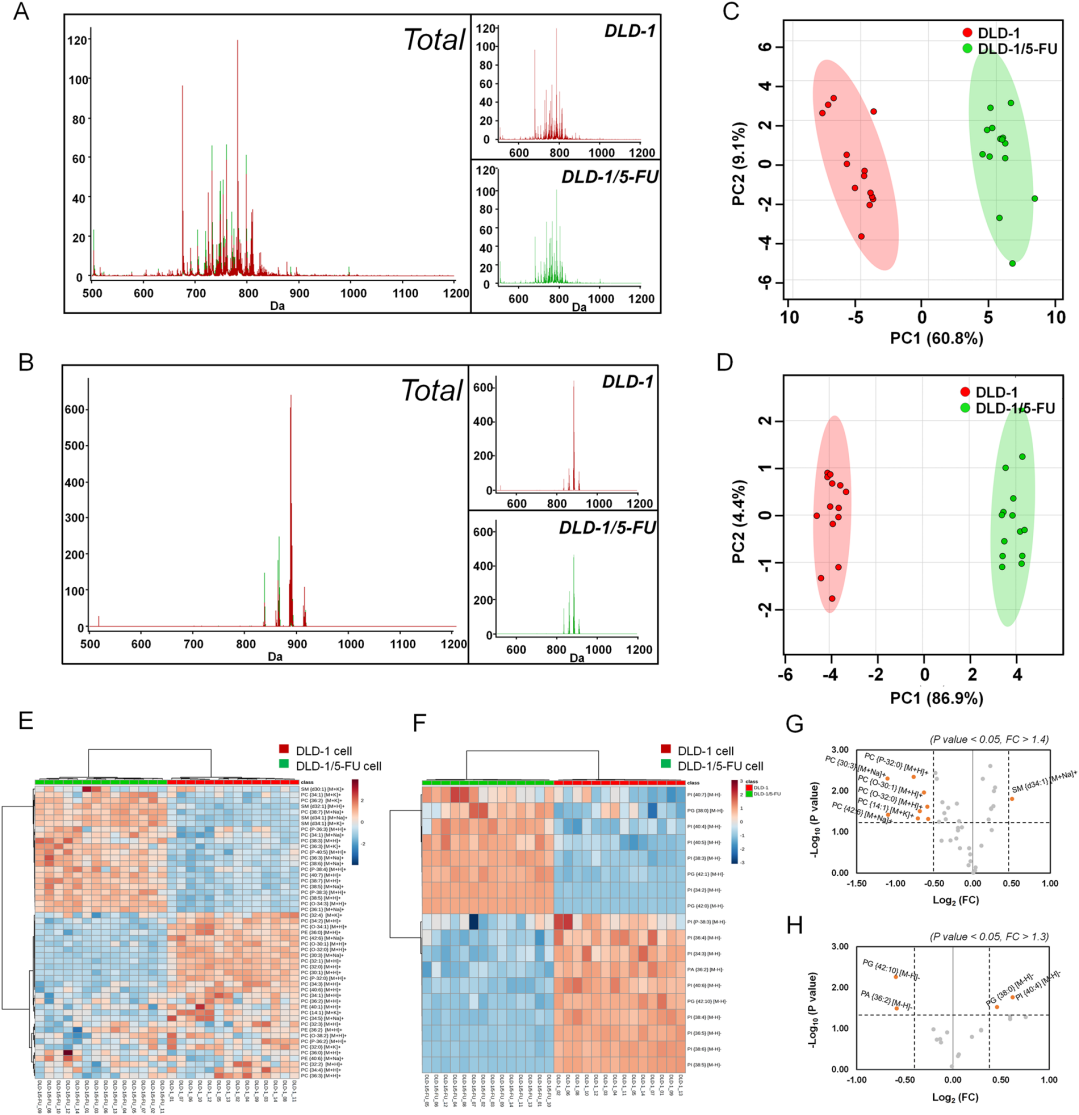
MALDI-TOF-MS lipidomic analysis of DLD-1 and DLD-1/5-FU cells. (**A**) Average chromatogram of DLD-1 (in red) and DLD-1/5-FU (in green) obtained with positive ion mode and (**B**) negative mode. (**C**) Principal component analysis (PCA) of the acquired MS spectra from DLD-1 (shown in red) and DLD-1/5-FU (shown in green) in positive and (**D**) negative modes. Hierarchical clustering of each sample data set showing differentially expressed lipids in (**E**) positive mode and (**F**) negative mode. Volcano plots display differentially expressed lipids in (**G**) positive and (**H**) negative mode, respectively **(**fold change > 1.4, p value < 0.05 in positive and fold change > 1.3, p value < 0.05 in negative mode).

The positive ion and negative ion MALDI-MS spectra of lipids acquired from DLD-1 (Red) and DLD-1/5-FU (Green) are shown in Fig. 2A,B. Most lipid peaks appeared in the scan ranges of m/z 600–900 in positive ion mode and m/z 800–950 in negative ion mode. We subjected the obtained mass spectral data to principal component (PCA) analysis to compare the general clustering trends of lipids between DLD-1 and DLD-1/5-FU (Fig. 2C,D). As seen from the PCA plot, significant different patterns in lipid compositions were observed between the two types of cells. From the MALDI-MS spectra of lipids, we could distinguish DLD-1 and DLD-1/5-FU cells with a 60.8% and 86.9% confidence interval in both positive and negative ion modes, respectively.

Each lipid was identified based on the search results of the precursor ion through Lipidomics Gateway (http://www.lipidmaps.org) and the MS/MS fragment ions^19,20^ through LIFT mode. Supplementary Fig. S1 shows representative MS/MS spectra of PC {34:1} [M + K]^+^ and PI {16:0/18:1} [M-H]^−^ obtained in both positive and negative ion modes in LIFT mode. In positive mode, Supplementary Fig. S1A depicts MS/MS spectrum of PC {34:1} [M + K]^+^ (m/z 798.6) showing fragment ions at m/z 184.1 corresponds to the choline head group and m/z 163.1 which is signature ion of the head group of PC with potassium adducts, symbolized [M + K]^+^. The negative ion MS/MS spectrum of m/z 861.6 (Supplementary Fig. S1B) displays product ions at m/z 241, corresponding to phosphatidylinositol (PI) head group. Additional fragment ions at m/z 255.3 and m/z 281.3 reflect the deprotonated fatty acyl chains of 16:0 and 18:1, respectively. Similarly, other identified lipids were also confirmed by MS/MS fragmentation patterns in LIFT mode (data not shown).

### Altered lipids in 5-FU resistant DLD-1 cells compared to sensitive cells in MALDI-MS analysis

A total of 42 lipids in positive ion mode and 18 lipids in negative ion mode was identified and quantified using Metaboanalyst 2.0 (Supplementary Tables S1 and S2). In particular, 35 species of PC, 4 species of PE, and 3 species of SM were identified in positive ion mode. In negative ion mode, 14 PI, 3 phosphatidylglycerol (PG), and 1 phosphatidic acid (PA) species were identified. Data of hierarchical clustering and heat mapping (Fig. 2E,F) show up-regulated (indicated in red) and down-regulated (indicated in green) patterns of lipids in both DLD-1/5-FU cells and DLD-1 cells. Next, we assessed differentially regulated lipids (DRLs) isolated from DLD-1/5-FU and compared them with those of DLD-1 using specific selection criteria (positive mode; fold change > 1.4, p value < 0.05 and negative mode; fold change > 1.3, p value < 0.05). A total of 9 DRLs (1 up-regulated and 8 down-regulated) was identified in positive mode. Of note, only SM species was significantly up-regulated in DLD-1/5-FU: SM {d34:1} [M + Na]^+^ (m/z 725.5). On the other hand, 8 PC species were significantly down-regulated in DLD-1/5-FU: PC {30:3} [M + Na]^+^ (m/z 722.5), PC {42:6} [M + Na]^+^ (m/z 884.6), PC {P-32:0} [M + H]^+^ (m/z 718.6), PC {14:1} [M + K]^+^ (m/z 504.2), PC {O-32:0} [M + H]^+^ (m/z 720.6), PC {O-30:1} [M + H]^+^ (m/z 690.6), PC {30:1} [M + H]^+^ (m/z 704.6) and PC {32:0} [M + H]^+^ (m/z 734.5). In negative ion mode, PI {40:4} [M − H]^−^ (m/z 913.6) and PG {38:0} [M − H]- (m/z 805.6) were increased, while PG {42:10} [M − H]^−^ (841.6 m/z) and PA {36:2} [M − H]^−^ (m/z 701.5) were decreased in 5-FU-resistant cells compared with sensitive cells. Volcano plots revealed that the DRLs in DLD-1/5-FU were largely classified into over-expressed or under-expressed clusters in both positive and negative ion modes (Fig. 2G,H).

### Quantification of sphingolipids in DLD-1/5FU and DLD-1 cells using LC-MRM-MS

MALDI-TOF analysis revealed an increase of sphingomyelin species. Therefore, we focused on how upregulation of sphingomyelin is related with 5-FU resistance in DLD-1 cells. To quantify SLs (including SM and Cer) that are important in apoptosis, cell proliferation, and drug resistance, we developed and optimized the MRM conditions for SM and Cer using LC-QqQ-MS. First, the lipid standards SM (d18:1–12:0), Cer (d18:1–12:0), dihydro-sphingomyelin (d18:1–12:0), dihydro-ceramide (d18:0–12:0), and ceramide-1-phosphate (d18:1–12:0) were used to optimize the MRM conditions for SLs. To quantify SL species in lipid samples, we detected their corresponding [M + H]^+^, [M + Na]^+^, and [M + K]^+^ ions by MS scan and later confirmed them with MS/MS of adducted ions of each lipid species under different collision energies. Based on the MS scan and MS/MS conditions, we set the MRM transitions (m/z value of precursor ion [Q1] > m/z value of product ion [Q3]) for each SL species as summarized in Supplementary Table S3.

In total, 55 SLs including 22 SMs, 11 dihydro-sphingomyelin (DHSM), 11 Cers, 7 ceramide-1-phosphate (C1P), and 1 each of sphingosine (d18:1) (So), sphinganine (d18:0) (Sa), sphingosine 1-phosphate (d18:1) (S1P), and sphinganine1-phosphate (d18:0) (Sa1P) were identified and quantified by MRM analysis (Supplementary Table S3). We further identified 22 SLs that were significantly differentially regulated between DLD-1/5-FU and DLD-1 cells (fold change > 1.5, p value < 0.05) (Table 1). The quantitative results demonstrated that SMs comprised the largest proportion of SLs, among which the C18 sphingoid base backbone was the dominant species. Among the 22 SMs, 16 showed an increase in DLD-1/5-FU cells compared to sensitive cells. In particular, 8 of the 9 SM species in DRLs increased significantly, which was similar to the MALDI-MS results. DHSM showed a slight increase with an average 1.18-fold change in DLD-1/5-FU cells, and 3 of the 5 DHSM increased, whereas the other 2 species decreased significantly.

**Table 1:** Differentially expressed sphingolipids between DLD-1/5FU and DLD-1 cells in LC-QqQ-MS based MRM quantification (Fold change > 1.5, p value < 0.05).

Category	Lipids	log2 (Fold change)	p value	Regulation
SM	SM(d18:1–20:2)	2.62	0.0012	Up
	SM(d18:0–20:0)	2.24	0.0071	Up
	SM(d18:1–22:4)	2.11	0.0219	Up
	SM(d18:1–22:2)	1.83	0.0014	Up
	SM(d18:1–20:0)	1.81	0.0001	Up
	SM(d18:1–22:1)	1.76	0.0002	Up
	SM(d18:1–22:3)	1.61	0.0001	Up
	SM(d18:1–20:3)	1.56	0.0013	Up
	SM(d18:1–24:0)	0.56	0.0021	Down
dihydro-SM	DHSM(d18:0–20:1)	1.75	0.0080	Up
	DHSM(d18:0–22:2)	1.74	0.0007	Up
	DHSM(d18:0–18:1)	1.53	0.0200	Up
	DHSM(d18:0–16:0)	0.64	0.0271	Down
	DHSM(d18:0–24:1)	0.54	0.0001	Down
Cer	Cer(d18:1–16:0)	0.64	0.0283	Down
	DHCer(d16:0–24:0)	2.13	0.0131	Up
C1P	Cer1P(d18:1–18:1)	1.85	0.0387	Up
	Cer1P(d18:1–22:0)	0.48	0.0356	Down
	Cer1P(d18:1–24:1)	0.39	0.0081	Down
	Cer1P(d18:1–24:0)	0.24	0.0264	Down
So	So(d18:1)	3.58	0.0006	Up
Sa	Sa(d18:0)	1.71	0.0154	Up

SM: Sphingomyelin, dihydro-SM: dihydrosphingomyelin, Cer: Ceramide, C1P: Ceramide-1-Phosphate, So: Sphingosine, Sa: Sphinganine

Interestingly, unlike the SM class, all 6 quantifiable species of Cer decreased except Cer (d18:1–24:1). In particular, the most dominant Cer (d18:1–16:0) in DLD-1 cells decreased by 0.64-fold and was identified as a marker lipid candidate for 5-FU resistance. In addition, we confirmed up-regulation of So (d18:1) by 3.58-fold and Sa (d18:0) by 1.71-fold.

### Quantification of enzymes in the sphingolipid pathway in DLD-1/5FU and DLD-1 cells by LC-MRM-MS

**Figure 3:**
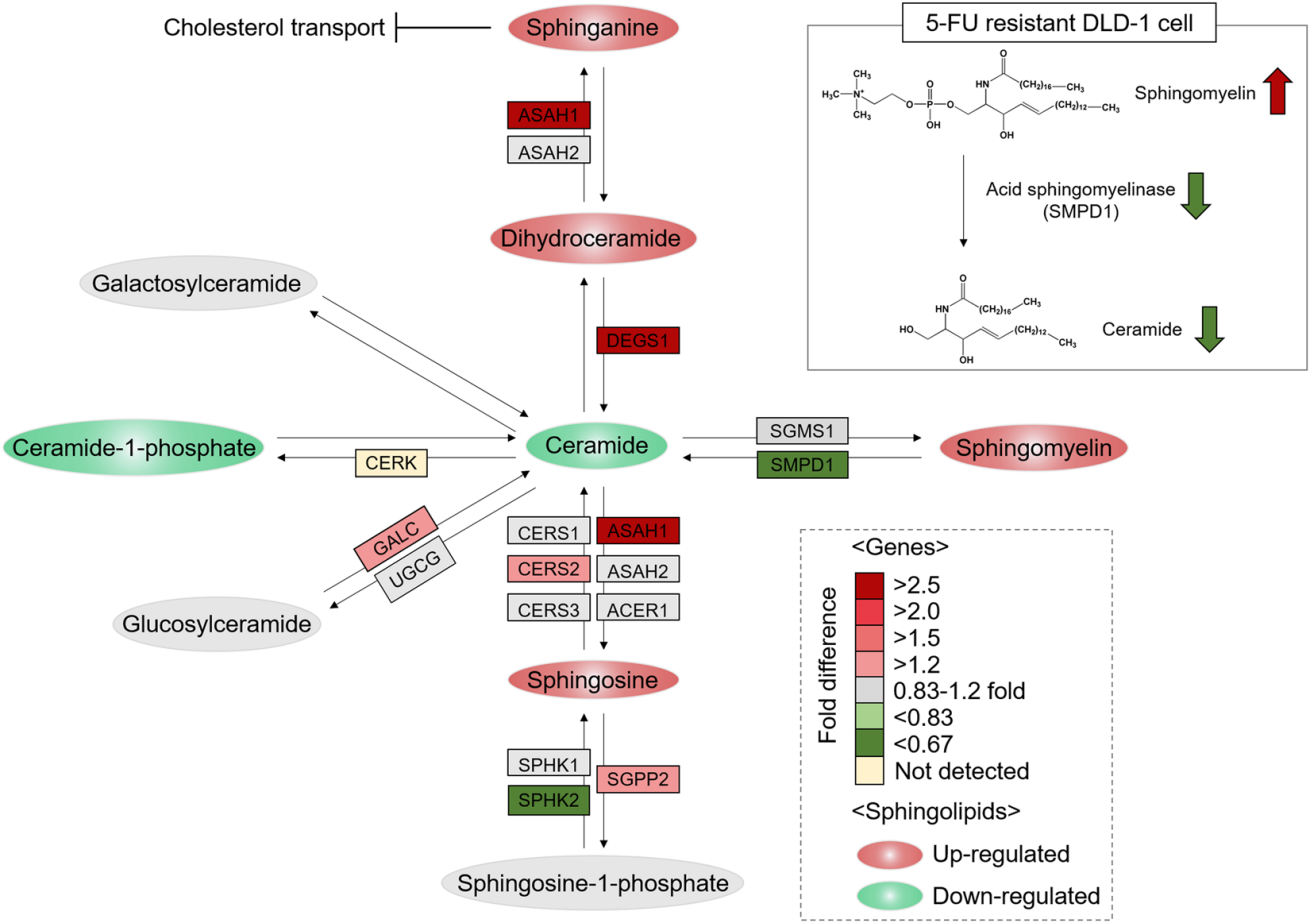
Quantitative results of sphingolipids and enzymes by LC-MRM-MS. Illustration of differences in the expression of sphingolipids and proteins in the sphingolipid metabolism pathway using MRM-based quantification. The abbreviations of proteins are shown in boxes with different colors to represent fold change in DLD-1/5-FU compared to DLD-1. Upregulated sphingolipids are shown in red circles, while downregulation is shown in green circles.

We investigated the relationship between 5-FU resistance and SLs through an integrated analysis of SLs in the SL metabolism pathway, as well as related enzymes. Prior to MRM quantification, 20 enzymes were confirmed as candidates including 75 distinct Q1 and 300 distinct Q3 transitions generated from SRMAtlas^21^, which provides definitive and verified peptide transitions and collision energy information optimized by quadrupole-based mass spectrometry (Supplementary Table S4). The detectable peptides were confirmed by an MRM transition test followed by MRM analysis in triplicate. The analysis showed that 5 enzymes—SMPD1, SPHK2, ASAH1, DEGS1, and GALC—were significantly changed in DLD-1/5FU compared to DLD-1 cells (p value < 0.05, fold change > 1.3) (Supplementary Table S5). As shown in Fig. 3, the enzyme ASAH1 (ceramidase family), whose product catalyzes the degradation of Cer into So(d18:1) and deacylates Cer into Sa(d18:0), was significantly up-regulated by 2.65-fold, as shown by increases in So(d18:1) and Sa(d18:0), respectively. Cer is further metabolized for the synthesis of galactosylceramide and glucosylceramide by UGCG (glucosylceramide synthase), which are precursors of lactosylceramide and ganglioside generation, respectively. However, these pathways were not altered significantly in 5-FU–resistant cells. Most importantly, the decrease of SMPD1 (acid sphingomyelinase; ASMase), which hydrolyzes SM to yield Cer in DLD-1/5FU cells, appeared to be related to increase of SM and decrease of Cer in lipidomic results.

### Gene silencing of SMPD1 induce acquisition of 5-FU resistance in DLD-1 cell

**Figure 4:**
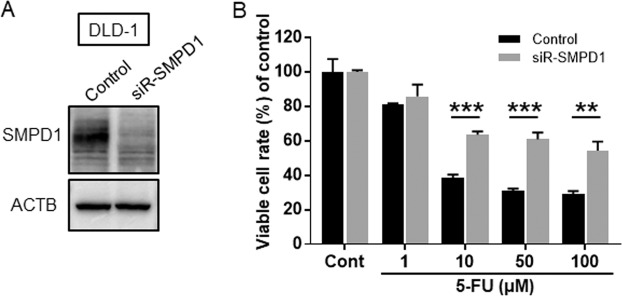
siR-SMPD1 quality check by western blotting analysis. (**A**) siR-SMPD1 quality check by western blotting analysis. The concentration of each siRNA was 2.5 nM. (**B**) The cell viabilities were examined after siR-SMPD1-treatment with different amounts of 5-FU. Results are presented as the mean ± SD; **p < 0.01; ***p < 0.001.

**Figure 5:**
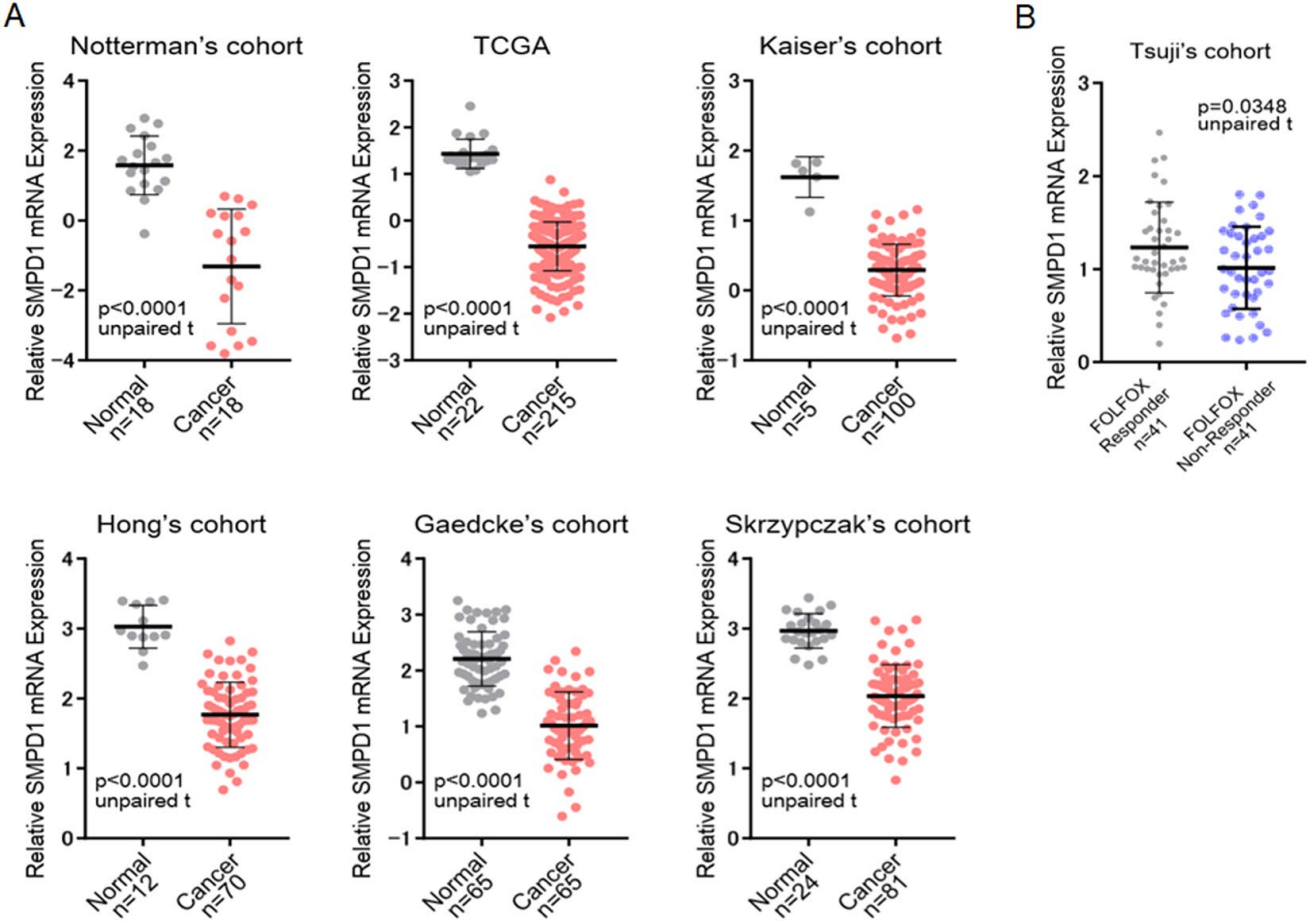
(**A**) The mRNA expression level of SMPD1 was investigated for several CRC cohorts. The n number of independent patient samples is indicated in each panel. An unpaired t-test was performed to examine the difference between the expression levels of SMPD1 mRNA in normal and CRC tissues. Error bars indicate standard deviations. (**B**) FOLFOX is 5-FU+ l-LV+L-OHP and is a standard chemotherapy regimen for CRC. SMPD1 mRNA expression level in FOLFOX responder and non-responder group from Tsuji’s cohort.

As confirmed in the MRM analysis, conversion of SM to Cer was attenuated in the 5-FU resistant cell line. These results strongly suggest that SMPD1-driven SM to Cer conversion is crucial in 5-FU resistance. SMPD1 is a major enzyme in the conversion of SM to Cer. To confirm whether the suppression of SMPD-1 expression was dependent on 5-FU resistance, the cell viability was examined by combined treatment with 5-FU after the gene-silencing for SMPD1 in DLD-1 parental cells. Western blot analysis showed that siRNA-SMPD1 reduced expression of SMPD1 as shown in Fig. 4A and Supplementary Fig. S2. Subsequently, when DLD-1 cells were treated with 1 to 100 μM 5-FU combined with siR-SMPD1, the growth inhibition by 5-FU was partly blocked in siR-SMPD1-treated DLD-1 parental cells as shown in Fig. 4B. Finally, we investigated the expression level of SMPD1 in CRC clinical specimens by clinical data sets (Oncomine database). As shown in Fig. 5A, down-regulation of SMPD1 was observed in tumor specimens compared with normal specimens in several data sets^22–27^. Importantly, Tsuji’s cohort indicated significant down-regulation of SMPD1 in non-responders to FOLFOX, a regimen that includes 5-FU^28^ (Fig. 5B). These findings suggested that down-regulation of SMPD1 is associated with the acquisition of 5-FU resistance in clinical CRC samples.

## Discussion

Since 5-FU has been used in cancer therapy, several important studies have been conducted to understand the mechanism underlying 5-FU resistance in CRC. Screening at the genomic and proteomic levels using microarray techniques and traditional molecular technologies offers an abundance of candidate targets that have key roles in 5-FU resistance^29–31^. However, outcomes from these strategies have not been enough to overcome 5-FU resistance in CRC. Thus, new approaches are needed to discover the mechanism of resistance to 5-FU.

In the present study, we carried out a sequential lipidomic analysis combined with a shotgun MALDI-TOF analysis and a LC-QqQ-MS-based MRM analysis to identify and quantify DRLs in 5-FU–resistant cells. Notably, our optimized sphingolipidomic analyses on DLD-1 and DLD-1/5-FU cells encompassed several definite SLs including SM, Cer, DHSM, So(d18:1), Sa(d18:0), and C1P, as well as the S1P(d18:1) and Sa1P(d18:0) classes. This is the first comprehensive lipidomic study on 5-FU–resistant CRC to date, as evidenced by the identification of up to 124 lipids including GPs and SLs. Furthermore, our study provides a broad understanding of the 5-FU–resistance mechanism by integrating SLs and the enzymes in the SL pathway.

We identified 60 lipids in both positive and negative ion modes of MALDI-MS analysis. In particular, we explored the alterations of several GP species including PC, PE, SM, PI, PG, and PA in both positive and negative ion modes. Among them, 8 PC species, PC {30:3} [M + Na]^+^, PC {42:6} [M + Na]^+^, PC {P-32:0} [M + H]^+^, PC {14:1} [M + K]^+^, PC {O-32:0} [M + H]^+^, PC {O-30:1} [M + H]^+^, PC {30:1} [M + H]^+^ and PC {32:0} [M + H]^+^ were significantly down regulated in DLD-1/5-FU cells (fold change < 0.71, p value < 0.05). Previous studies revealed that phospholipase D (PLD) enzymes catalyze the elimination of head groups of PC to generate PA, resulting in reduction of PC groups^32–34^. PLD enzymes have been implicated as key regulators in progression, tumorigenesis, and inhibition of apoptosis^35–38^ in CRC as well as in other types of cancers^39^. Although the present study noted the association between changes in sphingolipids and 5-FU resistance, further studies will be performed on phospholipids and their enzymes.

It is noted that MALDI-MS-based lipid profiling identified up-regulated SM species, SM {d34:1} [M + Na]^+^ (fold change > 1.4, p value < 0.05). However, the Cer species were not detected in MALDI-MS due to their low ionization efficiencies^40^.

To confirm the relationship between the alteration of SM and 5-FU resistance, we further quantified the SLs including SM, Cer, DHSM, So(d18:1), Sa(d18:0), C1P, S1P(d18:1), and Sa1P(d18:0) using the more sensitive and quantitative LC-QqQ-MS. We quantified 55 targeted SLs, as shown in Supplementary Table S3. In this study, the most significant alteration of SLs was the overall increase of SMs and decrease of Cers. Approximately 16 of the 22 SMs (73%) showed up-regulation, and 5 of the 6 Cers were significantly decreased in DLD-1/5FU cells (fold change > 1.5, p value < 0.05). The roles of membrane lipids such as PC, SM, Cer, and cholesterol in signaling and protein functions have been well investigated^41,42^. In addition, biophysical properties of cell membrane lipids have also been demonstrated to regulate apoptosis, proliferation, and drug resistance^43,44^. From the structural point of view, SMs have the highest affinity to cholesterol on account of the interaction between the C-3 hydroxyl group of cholesterol and the sphingosine moiety of SMs. This interaction can make the plasma membrane denser and more resistant to drug influx^10^. Thus, several studies have demonstrated that the increased levels of SM and cholesterol in drug-resistant cells could indicate that a less permeable cell membrane can significantly reduce diffusion of 5-FU^45,46^. In the current study, significant up-regulation of SMs in DLD-1/5-FU cells was observed and confirmed by both MALDI-MS and LC-MRM-MS analyses, which may reveal the key mechanism of 5-FU resistance.

In addition, we confirmed the overall decrease of Cer in DLD-1/5-FU cells compared to sensitive DLD-1 cells. It can be noted that Cer species are intracellular messengers that facilitate several signaling pathways that lead to cell cycle arrest^47^, apoptosis^48–50^, and autophagic responses^51,52^. In particular, C18-Cer induces cancer cell death and is an essential lipid for tumor suppression^53–55^. Interestingly, our study indicated that the decrease in Cer (d18:1–16:0) plays an important role in inhibiting 5-FU–induced apoptosis in CRC. The intracellular concentrations of SM and Cer are managed by regulating the SMPD1 level. Lower levels of intracellular Cer are maintained in various drug-resistant cells by either escalating SM level or hindering SM breakdown into Cer by controlling ASMase level^56^. V Gouazé *et al*. have demonstrated that ASMase is expressed in lower levels in doxorubicin-resistant breast cancer (T47D) cells compared to sensitive cells. This study revealed that doxorubicin sensitivity could be increased by the addition of exogenous Cer, which suggests that low ASMase level is critical for drug resistance^57^. In addition, Hao *et al*. investigated the significantly changed SL profiles in taxol-resistant ovarian cancer cells. They demonstrated that ASMase down-regulation is key to regulate formation of SMs and degradation of Cer in taxol-resistant ovarian cancer cells (A2780T)^58^. In our study, the reduction of SMPD1, which hydrolyzes SM to ceramide, was confirmed by MRM validation (Supplementary Table S5 and Fig. 3). Furthermore, the results of clinical data set analysis supported our findings derived by lipidomic approaches (Fig. 5). In this current study, we used the total lipids extracted from whole cells rather than plasma membrane or cytosol. Therefore, we were not able to interpret specific lipid-based localization. In order to analyze cytosolic lipid and membrane lipid separately, additional lipidomic analysis should be performed after separating each part. Therefore, in order to confirm more precise mechanism of sphinoglipids in 5-FU resistance, we plan to isolate plasma membrane and cytosol separately and subsequent lipidomic analysis will be performed in another study.

In conclusion, we successfully performed comprehensive profiling and quantification of GPs and SLs in both 5-FU–sensitive and –resistant CRC cells to investigate the mechanism for 5-FU resistance. The most significantly altered SL metabolism pathways in 5-FU–resistant cells were the up-regulation of SMs and down-regulation of Cers, which were controlled by SMPD1. We propose that DLD-1/5-FU cells can acquire resistance from inhibition of ceramide-caused apoptosis principally via the SM/Cer pathway, while SMPD1 was lower in 5-FU–resistant cells than in the sensitive cell line. Metabolic regulation of lipids and enzymes associated with the SL pathway is a potential target for treatment of 5-FU–resistant CRC. Based on the current lipidomic studies, modulation of SL metabolism may be a successful strategy to overcome 5-FU resistance and can provide a variety of therapeutic opportunities in the drug development process to aid CRC treatment.

## Methods

### Cell culture and establishment of DLD-1/5-FU cells

Human colorectal cancer DLD-1 cells were obtained from the Japanese Collection of Research Bioresources (JCRB) Cell Bank. DLD-1 and its 5-FU–resistant derivative DLD-1/5FU, which was acquired after selection by drug treatment, were cultured in RPMI-1640 medium supplemented with 10% (v/v) heat-inactivated fetal bovine serum (Sigma, St. Louis, MO, USA) under an atmosphere of 95% air and 5% CO2 at 37 °C. DLD-1/5-FU cells were established in our previous reports^59,60^. The cells were then harvested by trypsinization and sub-cultured at a density of 6 × 10^3^ cells/cm^2^. The media was changed after one day of sub-culturing, and all cell cultures were passaged again at 70–80% confluence. Cell line authentication was achieved by short tandem repeat (STR) analysis and testing for mycoplasma contamination. STR analysis was performed using primers of TH01, TPOX, vWA, amelogenin, CSF1PO, D16S539, D7S820, D13S317, D5S818, and D21S11 (GenePrint 10 System; Promega, Madison, WI). Testing for mycoplasma contamination was performed by exploiting the activity of certain mycoplasmal enzymes (MycoAlert mycoplasma detection kit; Lonza, Basel, Switzerland). The number of viable cells was determined by the trypan-blue dye exclusion test.

### Extraction of total lipids from cells

A total of 6 × 10^6^ cells was used for lipid analyses. Total lipids were extracted by the Bligh & Dyer method^61^. Briefly, each cell pellet was directly transferred into 3 mL of chloroform: methanol (1:2, v/v) in a 15 mL conical glass tube. Each sample was vortexed and sonicated for 10 min and allowed to cool on ice for about 10 min. Samples were then centrifuged at 2,500 g for 10 min. The bottom organic phase was dried in a speed vacuum. All experiments were performed in duplicate for reproducible results.

### MALDI-MS analysis of lipids

For MALDI-MS analysis of lipids, samples dissociated with approximately 10 µL of methanol/chloroform (70/30, v/v). In positive mode, 10 µL of the binary matrix solution (7 mg each of 2, 5-dihydroxybenzoic acid and α-cyano-4-hydroxycinnamic acid in 1 mL of 70% methanol plus 0.1% Trifluoroacetic acid) was mixed with lipid extracts. In negative mode, 10 µL of 9-aminoacridine (10 mg/mL; dissolved in isopropanol/acetonitrile (60/40, v/v)) was mixed with each sample^62,63^. Samples were spotted directly on a 384 target plate (Bruker Daltonics, Bremen, Germany) and dried in a desiccator for homogeneous crystallization to obtain reproducible results. Samples were spotted onto 14 wells for replicates for positive and negative ion modes, respectively. Consequently, we acquired 14 MS spectrum from biological two replicates and 7 instrumental replicates. MALDI-MS analysis was performed using an Ultraflex III TOF/TOF mass spectrometer (Bruker Daltonics, Bremen, Germany) equipped with a 200-Hz smart beam laser as an ionization source. All spectra were acquired with the mass range of 500–1200 m/z with the following parameters: delay: 180 ns; ion source 1: voltage, 25 kV, ion source 2: voltage, 21.65 kV; and lens voltage: 9.2 kV. External calibration was carried out using lipid-mixed calibration standards in ranges of m/z 674–834 Da (in positive ion mode) and m/z 564–906 Da (in negative ion mode). The structural identify of lipids was confirmed by LIFT (MS/MS) mode. MS/MS spectrum was acquired after manual monoisotopic selection with the following parameters: <LIFT mode condition> CID mode = false; PCIS mass limit = 2–4 Da; ion source voltage 1/2 = 8 kV/7.1 kV; LENS voltage = 3.6 kV; LIFT voltage 1/2 = 19 kV/4.3 kV in positive mode. CID mode = false; PCIS mass limit = 2–4 Da; ion source voltage 1/2 = 8 kV/7.1 kV; LENS voltage = 3.6 kV; and LIFT voltage 1/2 = 19 kV/4.2 kV in negative mode. Each lipid was assigned based on the LIPID MAPS classification system.

### Pre-processing of MALDI-MS data

The MALDI quant package in R was used for pre-processing^64^. The transformation to a square root and smoothing with a moving average algorithm were applied all the features in spectrum for variance stabilization. The spectrum background was estimated with a statistics-sensitive nonlinear iterative peak-clipping algorithm and used for baseline correction. The peak intensities of each features were normalized using a probabilistic quotient normalization method^65^. Each spectrum was normalized to a reference spectrum, which was the median spectrum of all samples. Briefly, the normalization procedure was as follows: (1) normalization of all spectra to TIC, (2) calculation of the reference spectrum, (3) for each spectrum, calculation of the quotients of the intensities of the spectrum with those of the reference spectrum, (4) calculation of the median of these quotients, and (5) division of all intensities of the spectrum by the median of the quotients calculated at step 4. The Signal-to-Noise threshold greater than 3 was applied to considered peaks.

### Statistical analysis of MALDI MS data

For relative quantification, the following statistical analyses were carried out using MetaboAnalyst 2.0, web-based software for quantitative data analysis^66^. First, missing values obtained from a pre-processing procedure were replaced by half of the minimum positive value. The intensity values of each peak across multiple spectra were mean-centered and divided by the standard deviation. Principal component analysis (PCA) was carried out to classify the variance among samples between DLD-1 and DLD-1/5-FU. The lipids that were regulated differentially were identified using the following criteria: (1) averaging seven instrumental replicates to one representative value (mean), (2) p values from t-test are less than 0.05 and (3) relative fold changes are larger than 1.4 and 1.3 in positive and negative ion mode, respectively. Hierarchical clustering of DRLs was performed using “Euclidean distances” and “ward” linkage.

### MRM quantification of sphingolipids using LC-QqQ-MS analysis

Quantification of SLs including SM, Cer, DHSM, C1P, So(d18:1), Sa(d18:0), S1P(d18:1), and Sa1P(d18:0) was performed using a triple quadrupole (QqQ) mass spectrometer (6490 series, Agilent Technologies, Wilmington, DE, USA) coupled to a 1200 series HPLC system (Agilent Technologies, Wilmington, DE, USA). Lipids were separated on a Hypersil GOLD column (2.1 × 100 mm ID; 1.9 μm, Thermo Fisher Scientific, USA) with the temperatures of the column oven and sample tray were set to 40 °C and 4 °C, respectively. The mobile phase A composition was acetonitrile:methanol:water mixture (19:19:2) with 20 mmol/L ammonium formate and 0.1% (v/v) formic acid, and B composition was 2-propanol with 20 mmol/L ammonium formate and 0.1% (v/v) formic acid. Sphingolipids were separated with 30-minutes nonlinear gradient as follow: holding the solvent mixture steady 5% solvent B for 5 min, followed by a first linear gradient to 30% solvent B for 10 min, a second linear gradient to 90% solvent B for 7 min, an isocratic elution for washing step to 90% solvent B for 3 min, and a third linear gradient to 5% solvent B for 1 min. The column was equilibrated with 5% solvent B for 4 min at a flow rate of 250 μL/min. The electrospray (ESI) MS method was used to analyze lipids, and all acquisition method parameters were set as follows: capillary voltage: 3500 V in positive mode and 3000 V in negative mode, sheath gas flow: 11 L/min (UHP nitrogen) at 200 °C, drying gas flow: 15 L/min at 150 °C, and nebulizer gas flow at 25 psi. MS/MS collision energies, multiple reaction monitoring (MRM) conditions. For relative quantification, respective internal standards (IS) were used to assign the specific retention time (RT) of each SL group. After that, the peak area was extracted using Skyline software to quantify each lipid species, and then data normalization (lipid species peak area/IS peak area) was performed for lipid quantifications. All experiments performed in triplicates.

### Verification of sphingolipid-related enzymes by MRM analysis

The LC-QqQ-MS–based MRM verification for enzymes in the SL metabolism pathway was performed using 100 μg protein samples from DLD-1 and DLD-1/5-FU cells in triplicate. The pair of m/z values that are isolated in Q1 and Q3 and optimized collisional energy were referred to the Human SRMAtlas database^21,67^. Peptides were separated on an Agilent 1290 LC RP-HPLC equipped with a RP-HPLC column (150 × 2.1 mm ID, Agilent Zorbax Eclipse Plus C18 Rapid Resolution HD, 1.8 μm particles), and an Agilent 6490 triple-quadrupole mass spectrometer using a gradient from 5% to 40% solvent B (90% ACN, 0.1% formic acid) over 40 min. To gain a sufficient number of data points, the dwell time for each transition was determined between 6.55 and 248.88 ms, with transitions being the maximum number monitored in a given 1000-ms cycle. The areas were extracted using Skyline 3.7 ver. Savitzky-Golay smoothing filter was applied to improve the quality of the chromatograms. Peptide areas were normalized to that of a ß-galactosidase peptide (APLDNDIGVSEATR, 729.36 m/z (Q1) → 563.28 m/z (Q3)) to correct for experimental variation. The best transition was selected on the basis of intensity and consistency for quantification. Independent t-test analysis was conducted to determine the significance of target enzymes^68^.

### Western blot analysis

The reagents used were as follows: RIPA buffer for preparing cell lysates (Thermo Fisher Scientific Inc., Waltham, MA, USA), Protease Inhibitor Cocktail (Sigma-Aldrich Co. LLC, St. Louis, MO, USA), polyacrylamide gels for SDS-PAGE (Wako Pure Chemical Industries, Ltd. Osaka, Japan), PVDF membrane (Bio-Rad Laboratories, Inc., Hercules, CA, USA), PVDF Blocking Reagent for Can Get Signal (TOYOBO CO., LTD., OSAKA, JAPAN), and Luminata Forte Western HRP Substrate (Millipore Corporation, Billerica, MA, USA). The immunoblots were visualized by Fusion-FX7 (Vilber Lourmat, Marne-la-Vallée, France). Primary antibodies used were as follow anti-SMPD1 (Proteintech Group, Inc., Chicago, USA, 14609–1-AP) and anti-β-actin (Sigma-Aldrich Co. LLC, A2228). Also, β-actin was used as an internal control.

### Gene silencing experiments

siRNAs for SMPD1 (Invitrogen, Carlsbad, CA, USA) were used for the transfection of the cells, which was achieved by using cationic liposomes, Lipofectamine RNAiMAX (Invitrogen), according to the manufacturer’s protocol. Silencer Select Negative Control #1 siRNA (Ambion, Inc. Foster, CA, USA) was used as control. siR-SMPD1 was designed by BLOCK-iT RNAi Designer and the sequence is 5′-AUCAAGAGCCAGAAGUUCUCACGGG-3′. The effects manifested by the introduction of siRNAs into the cells were assessed at 48 h after the transfection. DLD-1 cells were seeded in 96-well plates at a concentration of 0.5 × 10^5^ per well (10–30% confluence) on the day before the treatment. We treated with 5-FU 24 h after transfection with siR-SMPD1 (2.5 nM) and the effects were assessed at 48 h after the treatment with 5-FU. The number of viable cells was determined by performing the MTT assay. MTT reagent, 3-(4, 5-dimethylthiazol-2-yl)-2, 5-diphenyltetrazolium-bromide), was purchased from (Sigma-Aldrich). MTT (0.5 mg/ml) was added to each well (10 μl/well) and after incubation for 2.5 hr at 37 °C, Supernatant were removed and. Then, Dimethyl sulfoxide (DMSO) was added to each well (200 μl/well). Absorbance at 540 nm was measured by SH-1000Lab microplate reader (Corona Electric Co., Ltd., Ibaraki, Japan).

### Clinical data set analysis

Oncomine was used in our clinical data set analysis (https://www.oncomine.org/resource/login.html). The mRNA expression levels were examined in each cohort study. The detailed information from each cohort study was cited as a reference.

## Supplementary information

Supplementary Information.

Supplementary Information 2.

Supplementary Information 3.

Supplementary Information 4.

Supplementary Information 5.

Supplementary Information 6.
