# Supplementary material for: Comparative lipidomics of 5-Fluorouracil–sensitive and –resistant colorectal cancer cells reveals altered sphingomyelin and ceramide controlled by acid sphingomyelinase (SMPD1)
Source: Sci Rep. 2020 Apr 9;10:6124. doi: 10.1038/s41598-020-62823-0 (PMC7145850; doi:10.1038/s41598-020-62823-0)
Supplement: Supplementary file 6 — Supplementary Information 6. [file 41598_2020_62823_MOESM6_ESM.pdf]

Comparative lipidomics of 5-Fluorouracil–sensitive and –resistant colorectal cancer cells reveals altered sphingomyelin and ceramide controlled by acid sphingomyelinase (SMPD1)

Jae Hun Jung[1], Kohei Taniguchi[3], Hyeong Min Lee[1], Min Young Lee[4], Raju Bandu[1], Kazumasa Komura[3], Kil Yeon Lee[5], Yukihiro Akao[6,**], and Kwang Pyo Kim[1,2,*]

[1]Department of Applied Chemistry, Institute of Natural Science, Global Center for Pharmaceutical Ingredient Materials, Kyung Hee University, Yongin, Republic of Korea. [2]Department of Biomedical Science and Technology, Kyung Hee Medical Science Research Institute, Kyung Hee University, Seoul, Republic of Korea. [3]Translational Research Program, Osaka Medical College, 2-7 Daigaku-machi, Takatsuki, Osaka 569-8686, Japan. [4]Institute for Systems Biology, Seattle, WA, USA. [5]Department of Surgery, College of Medicine, Kyung Hee University, Seoul, Republic of Korea. [6]United Graduate School of Drug Discovery and Medical Information Sciences, Gifu University, 1-1 Yanagido, Gifu 501-1193, Japan.

* Corresponding author. Department of Applied Chemistry, Institute of Natural Science, Global Center for Pharmaceutical Ingredient Materials, Kyung Hee University, Yongin, Republic of Korea.

** Corresponding author. United Graduate School of Drug Discovery and Medical Information Sciences, Gifu University, 1-1 Yanagido, Gifu 501-1193, Japan.

E-mail address: kimkp@khu.ac.kr (K.P.Kim), yakao@gifu-u.ac.jp (Y. Akao)

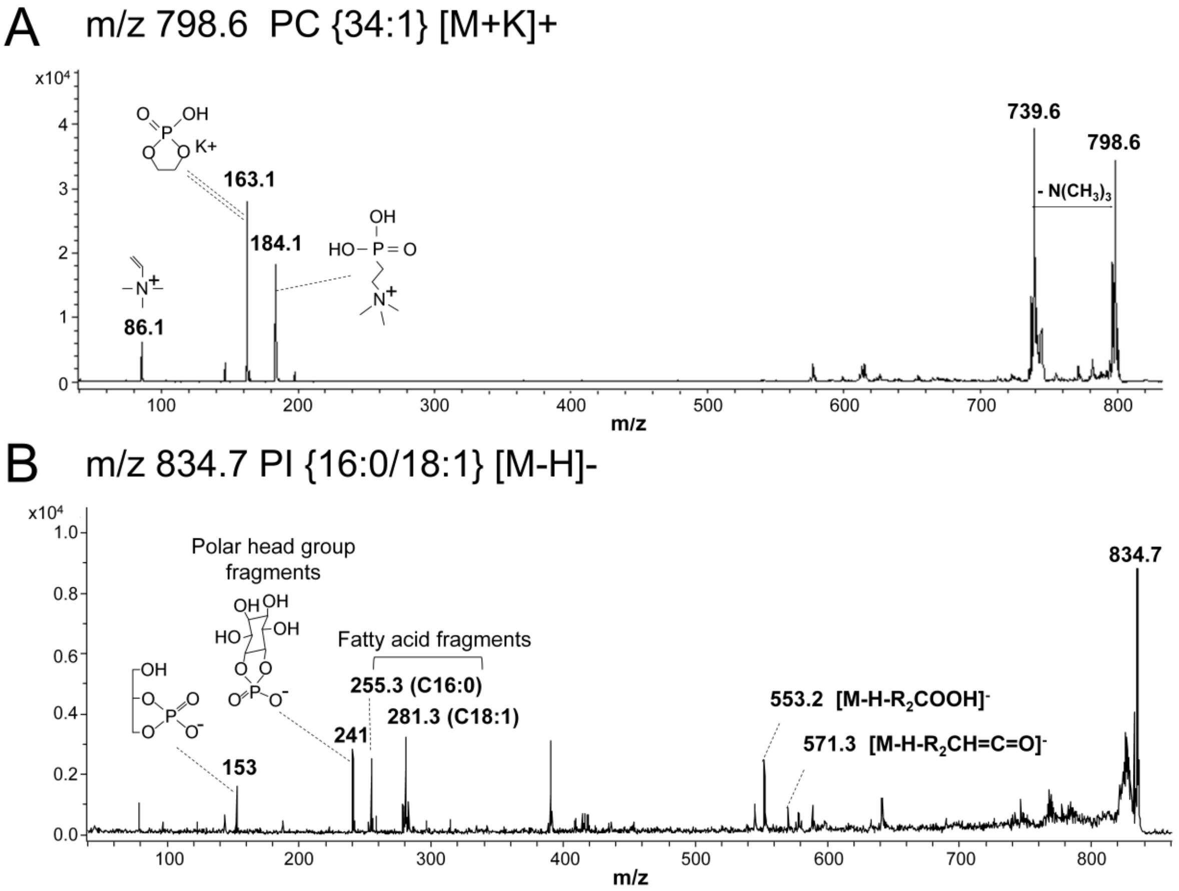

Supplementary Figure S1. Representative MS/MS spectra using the LIFT technique for phospholipid species. (A) MS/MS spectra of PC {34:1} [M+K]+ at m/z 798.6 in positive ion mode and (B) PI {16:0/18:1} [M-H]- at 834.7 m/z in negative ion mode.

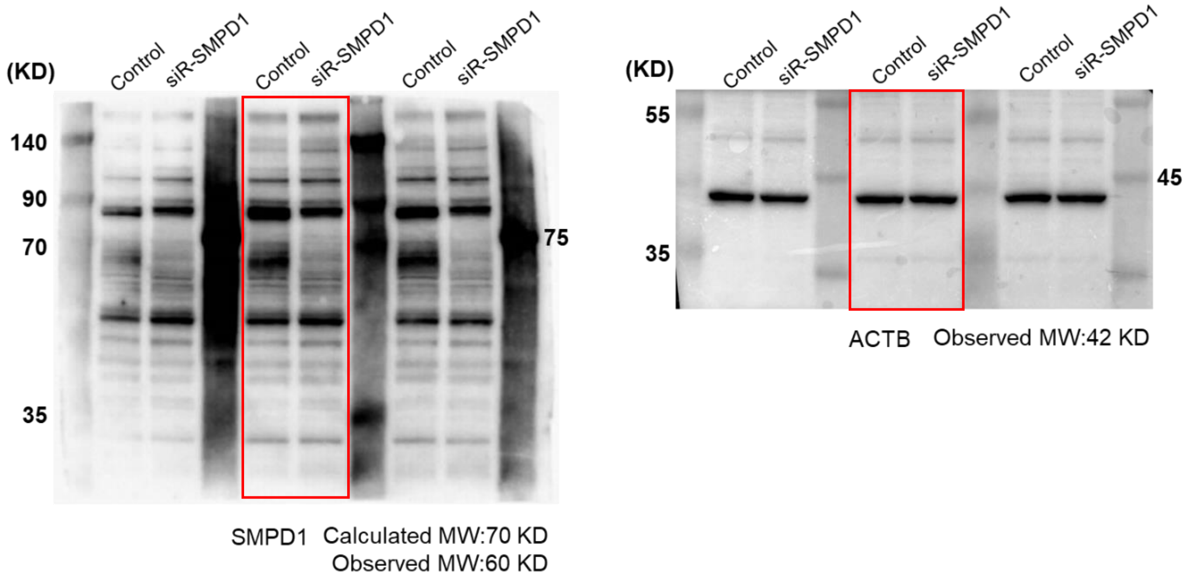

Supplementary Figure S2. Full blots of figure 4A. Red box indicated the data used as a figure. The expressions of SMPD1 and ACTB were examined in same gel. The methods regarding gene-silencing and western blot experiments were described in method parts of main manuscript.
